# Hypoxia suppressed the Siglec-5 signaling in TAMs via modulating the balance of SHP2/SYK activation in hepatocellular carcinoma

**DOI:** 10.1038/s41598-025-14040-w

**Published:** 2025-08-26

**Authors:** ShuBin Luo, JiaXi Mao, Lei Zhang, JiaJing Duan, GuoYou Xue, Qian Xu, ETao Yu

**Affiliations:** 1 Department of General Surgery (Section 1), The First People’s Hospital of Jinghong, Xishuangbanna Dai Autonomous Prefecture, Yunnan Province China; 2 Department of Liver Surgery and Organ Transplantation, Changzheng Hospital, Naval Medical University, Shanghai, 200003 China; 3 Department of General Surgery, Ruijin Hospital, Shanghai Jiaotong University School of Medicine, Shanghai, 20001 China; 4 The People’s Hospital of Xishuangbanna Dai Autonomous Prefecture Medical Laboratory, 4 Gai Lan Nan Road, Jinghong Street, Jinghong City, Xishuangbanna Dai Autonomous Prefecture, 666100 Yunnan Province China

**Keywords:** Siglec-5, ROS, SHP2/SYK, Hepatocellular carcinoma, Cancer prevention, Cancer therapy, Cancer, Molecular biology

## Abstract

**Objective:**

To investigate the effects of Siglec-5 on hepatocellular carcinoma and the mechanism of action. The interactions and expression changes between Siglec-5 and Siglec-14 not only affect immune cell function, but may also influence tumor progression. A deeper understanding of the mechanisms regulating their balance could provide new insights and strategies for hepatocellular carcinoma treatment.

**Methods:**

A cell co-culture model was established. Western blotting was used to detect protein expression in different groups. The CCK-8 assay was employed to observe the proliferation of HepG2 hepatocellular carcinoma cells, and the Transwell assay was used to examine their migration. Tumorigenic capacity of HepG2 cells detected by subcutaneous transplantation tumor assay in nude mice.

**Results:**

Overexpression of Siglec-5 under hypoxic conditions resulted in increased levels of SHP2 and arginase-1 proteins and decreased levels of P-SYK, IL-1β, and TNFα proteins. Addition of SHP2 inhibitor under hypoxic conditions or Siglec-5 overexpression resulted in increased expression of P-SYK and NOX4 proteins and decreased levels of arginase-1. When Siglec-5 expression was inhibited under hypoxic conditions, the levels of P-SHP2 and arginase-1 were decreased, whereas the levels of P-SYK, IL-1β, and TNFα were increased. The expression of P-SYK, NOX4, IL-1β, and TNFα was decreased, whereas the levels of arginase-1 were increased after the use of SYK inhibitors under hypoxic conditions that inhibited Siglec-5 expression. The proliferation, migration and tumorigenicity of HepG2 cells were increased when Siglec-5 was overexpressed under hypoxic conditions, while the proliferation, migration and tumorigenicity of HepG2 cells were decreased when Siglec-5 was inhibited under hypoxic conditions.

**Conclusion:**

Hypoxia suppressed the Siglec-5 signaling in TAMs via modulating the balance of SHP2/SYK activation in hepatocellular carcinoma.

**Supplementary Information:**

The online version contains supplementary material available at 10.1038/s41598-025-14040-w.

## Introduction

Hepatocellular carcinoma is one of the most common malignant tumors globally, with particularly high incidence and mortality rates in East Asia. Despite the improvements in patient outcomes with current treatments, the high recurrence rate and drug resistance of hepatocellular carcinoma remain significant challenges. Therefore, exploring new biomarkers and therapeutic targets is crucial for enhancing the diagnosis and treatment of hepatocellular carcinoma. Among the numerous potential targets, members of the SIGLEC (Sialic Acid-binding Immunoglobulin-like Lectin) family have garnered increasing attention. Siglec-5, as a key member of the SIGLEC family, has not yet been fully elucidated in terms of its role and mechanisms in hepatocellular carcinoma, thus warranting further investigation^1^.

Siglec-5 is a membrane protein expressed on immune cells and regulates biological processes through interactions with cell surface carbohydrates. Research indicates that Siglec-5 exhibits specific expression patterns and functions in certain types of cancer; however, its role in hepatocellular carcinoma remains to be further studied. Recent studies have found that Siglec-5 is expressed at higher levels in hepatocellular carcinoma cells and tissues, suggesting its potential involvement in the development and progression of hepatocellular carcinoma^2,3^. Notably, all known primate Siglec-5 genes are undergoing partial gene conversions with the adjacent Siglec-14 gene. Siglec-14, another member of the SIGLEC family, is closely related to immune and inflammatory responses. Research has shown that Siglec-14 can modulate immune cell functions through interactions with its ligands, thereby affecting the tumor immune microenvironment^4^. SYK (Spleen Tyrosine Kinase) is an important tyrosine kinase involved in regulating various cell signaling pathways, including those related to immune responses and inflammation^5^. Siglec-14 regulates immune cell activity through interactions with SYK, thereby influencing tumor progression^6,7^. Particularly in hepatocellular carcinoma, the Siglec-14/SYK signaling pathway may participate in the progression of hepatocellular carcinoma by modulating inflammatory responses and the immune microenvironment.

We hypothesize that the interaction between Siglec-14 and Siglec-5 forms a key regulatory axis that can regulate the progression of hepatocellular carcinoma. Specifically, elevated Siglec-5 expression may promote tumor progression by remodeling the immunosuppressive microenvironment. In addition, Siglec-14 may act as a mechanism of interference to inhibit the oncogenic effects of Siglec-5. To test this hypothesis, the present study aims to systematically characterize the expression pattern of Siglec-5 and its molecular mechanisms in HCC, to determine the functional effects of manipulating Siglec-5 on tumor behavior and immune regulation, and to experimentally validate the signaling pathway underlying the effects of Siglec-5 on hepatocellular carcinoma and assess the therapeutic potential of targeting this signaling pathway. These studies will establish the biological significance of Siglec-5 in the pathogenesis of HCC and identify new strategies for regulating the immune microenvironment.

## Materials and methods

### Source of experimental cell lines

Human monocyte line THP-1 and hepatocellular carcinoma cell line HepG2 were obtained from the Shanghai Cell Bank, Chinese Academy of Sciences.

### Experimental materials

Siglec-5 overexpression lentiviral vector plasmids, Siglec-5 knockdown (shRNA) lentiviral vector plasmids, and corresponding empty lentiviral vector plasmids were provided by Beijing Qunshi Jin Biotechnology Co., Ltd. Arginase-1, IL-1β, and TNFα enzyme-linked immunosorbent assay (ELISA) kits were purchased from Wuhan Elabscience Biotechnology Co., Ltd. H_2_O_2_ and SHP1/SHP2 inhibitor NSC87877 were obtained from Beijing BioLaureate Technology Co., Ltd. SYK inhibitor R406 was acquired from Yisheng Biotechnology (Shanghai) Co., Ltd. Transwell chambers were purchased from MERCK.

### Bioinformatics analysis

RNA sequencing data from the TCGA-LIHC project were downloaded and processed using the STAR pipeline from the TCGA database (https://portal.gdc.cancer.gov/)^8,9^. The ssGSEA algorithm from the R package GSVA[1.46.0] was employed to assess Siglec-5 immune infiltration using macrophage markers provided in the Immunity article. Data set GSE39791 from the GEO database, containing 144 samples (72 from healthy individuals and 72 from hepatocellular carcinoma patients), was analyzed. A threshold of |log2 Fold Change| > 2 and *p* < 0.05 was set. Missing values in the data were imputed using the impute package, and data were further normalized using the normalizeBetweenArrays function from the limma package. Box plots were generated using the ggplot2 package. RNA sequencing data from TCGA-LIHC were used to validate differential gene expression. Gene Ontology (GO) and Kyoto Encyclopedia of Genes and Genomes (KEGG) pathway enrichment analyses were conducted using the ClusterProfiler package. GO analysis included Cellular Component (CC), Molecular Function (MF), and Biological Process (BP), while KEGG pathway analysis aimed to identify key biological pathways. Correlation scatter plots were generated to analyze the relationships between differential genes^10^.

### Cell co-culture and grouping

THP-1 and HepG2 cells were cultured in RPMI 1640 medium supplemented with 10% fetal bovine serum. THP-1 was induced into macrophages using 100 ng/ml Phorbol 12-myristate 13-acetate (PMA). And it was indirectly co-cultured with HepG2 cells. HepG2 cells exhibited stable in vitro growth with a consistent doubling time and reproducible growth curve. As long as the appropriate culture conditions were provided, HepG2 cells could proliferate continuously and stably, providing a large number of homogeneous cell samples for the experiments, which ensured the reliability and reproducibility of the experimental results. Moreover, HepG2 cells required relatively low culture conditions and were easy to be cultured in the laboratory environment. Common culture medium, such as DMEM medium, with appropriate amount of serum and antibiotics, could be used to meet their growth requirements. In gene function research and gene therapy research, it was needed to introduce exogenous genes into the cells. HepG2 cells could accept the introduction of exogenous DNA, RNA or protein relatively easily with high transfection efficiency, which was convenient for researchers to carry out gene manipulation and functional verification.

In the first experiment, THP-1 cells were randomly divided into four groups: normoxia group (Group A), hypoxia group (Group B), normoxia + H_2_O_2_ group (Group C), and hypoxia + H_2_O_2_ group (Group D). The concentration of H_2_O_2_ used was 400 µmol/L.

In the second experiment, THP-1 cells were randomly divided into six groups: normoxia group (Group ①), hypoxia group (Group ②), hypoxia + Siglec-5 overexpression (OE) group (Group ③), hypoxia + Siglec-5 OE + NSC87877 group (Group ④), hypoxia + Siglec-5 knockdown (KD) group (Group ⑤), and hypoxia + Siglec-5 KD + R406 group (Group ⑥). The hypoxia group was incubated in a triple gas incubator at 1% O_2_. The dosage of NSC-87877 was 0.25 µM, and the dosage of R406 was 30 nM. The Siglec-5 OE group used the LV-SIGLEC5-EF1α-GFP virus (titer ≥ 1 × 10^8^ TU/mL), the Siglec-5 KD group used the LV-shSIGLEC5-U6-Puro virus with the target sequence 5’-GCAAGTTCCTGATCTACAA-3’, and the negative control group (NC) used the empty vector LV-Control-GFP/Puro virus. During infection, viral particles (MOI = 20) and 8 µg/mL polyvinylamine (Polybrene) were added to each well to enhance infection efficiency. Centrifugal infection was then performed (1000 × g, 32 °C, 90 min), followed by incubation of the cells at 37 °C in a 5% CO_2_ incubator for 48 h. Following viral transduction, stable transduced cell lines were selected using 2 µg/mL puromycin. Subsequently, the treated THP-1 cells were co-cultured with HepG2 cells via indirect co-culture. Transwell chambers were placed in culture plates, with HepG2 cells seeded in the upper chamber and THP-1 cells seeded in the lower chamber. After co-culturing for 48 h, subsequent experiments were conducted.

### Immunofluorescence

THP-1 cells from the normoxia (Group A), hypoxia (Group B), normoxia + H_2_O_2_ (Group C), and hypoxia + H_2_O_2_ (Group D) groups were fixed with 4% paraformaldehyde (PFA) for 30 min. Cells were permeabilized with 0.1–0.5% Triton X-100 to allow antibody entry. Blocking was performed with PBS containing BSA to reduce non-specific binding, typically for 1 h at room temperature. Primary antibodies against Siglec-5 and phosphorylated SHP2 (Y542 site) were used. Primary antibody incubation was done overnight at 4 °C or 1–2 h at room temperature. After washing with PBS to remove unbound primary antibodies (3 washes, 5–10 min each), secondary antibodies labeled with different fluorescent dyes (e.g., Alexa Fluor 488 and Alexa Fluor 594) were used. Secondary antibody incubation was for 1 h at room temperature. Samples were washed again with PBS to remove unbound secondary antibodies, and nuclei were stained with DAPI for 5–10 min to avoid over-staining. Fluorescence microscopy was used to observe the samples, with appropriate filters chosen to detect the signals for Siglec-5 and P-SHP2^11^.

### Western blot

THP-1 cells from the four and six groups in the logarithmic growth phase were plated at 5 × 10^5 cells per well in a 6-well plate. Cells were lysed with 400 µl of lysis buffer containing PMSF, and protein concentration was measured using a BCA kit. Proteins were separated by SDS-PAGE, transferred to PVDF membranes, and blocked with 5% non-fat dry milk for 1 h at room temperature. Membranes were incubated with primary antibodies (1:1000) overnight at 4 °C, followed by washing with TBST. Membranes were then incubated with HRP-conjugated secondary antibodies (1:10000) for 1 h at 25 °C. ECL substrate was used for chemiluminescence detection, and images were captured. Relative expression levels of proteins and the housekeeping protein GAPDH were analyzed using Image J software^12^.

### ELISA

Cells in the HepG2 and THP-1 co-culture system were cultured to an appropriate density. The supernatant was collected by centrifugation (4 °C, 3000 rpm, 10 min) for ELISA detection. Reagents and standards were prepared according to the kit instructions. The ELISA plate was coated with capture antibodies and incubated overnight at 4 °C. Blocking was performed with PBS containing non-fat dry milk for 1 h at room temperature. Diluted cell supernatant was added to the wells and incubated with capture antibodies. Incubation times and conditions were followed as per the kit instructions^13^.

### CCK-8 assay

HepG2 cells from the six groups were seeded into a 96-well plate at 80–90% confluence. After 6 h of incubation at 37 °C with 5% CO_2_, 10 µL of CCK-8 working solution was added to each well. Absorbance at 450 nm was measured after 48 h^14^.

### Transwell migration assay

200 µl of DMEM medium was added to the upper chamber of Transwell inserts (8 mm membrane diameter), and approximately 2 × 10^5 HepG2 cells from the six groups were seeded. Three replicates were performed for each group. DMEM medium containing 10% FBS was added to the lower chamber to attract cells from the upper chamber through the membrane. The Transwell inserts were placed in a 37 °C incubator for 24–48 h. After incubation, inserts were fixed with formaldehyde and air-dried. Cells were stained with 0.1% crystal violet for 20–30 min. Five random fields of view were selected under a microscope to count and photograph the migrated tumor cells^14^.

### Subcutaneous transplantation of tumors in nude mice

Twenty-four BALB/c-nu nude mice (6 weeks old, male, 20 ± 1 g) were provided by Henan SCBS Biotechnology Co. The nude mice were divided into Siglec-5 overexpression negative control (OE-NC) group, Siglec-5 overexpression (OE) group, Siglec-5 knockdown negative control (KD-NC) group, and Siglec-5 knockdown (KD) group. They were kept under standard laboratory conditions (25 ± 1 °C, 50% humidity, 12 h light/12 h dark cycle) and provided with adequate food and water. Siglec-5 knockdown and overexpression as well as negative control lentivirus infection of THP-1 cells from Siglec-5 KD and Siglec-5 OE groups were performed using lipofectamine 3000 reagent to establish stable cell lines. HepG2 cells and THP-1 cells in the logarithmic growth phase of each group were mixed in a 1:1 ratio, and the cell density was adjusted to 1 × 10^7^ cells/mL and added into 0.5 mL matrix gel. The cell mixture was injected subcutaneously into the back of nude mice, and the tumor size (V = 0.52 × L × W²) was measured and recorded. Twenty days after inoculation, the nude mice were gas-induced anesthetized with 3% isoflurane, and after anesthesia was achieved, the concentration was maintained at 1.5%, and then the tumors were harvested. The experiment was terminated immediately when the nude mice developed uncontrollable pain. Nude mice were euthanized at a flow rate of 30% solvent CO_2_ per minute. The design and implementation of animal experiments in this study were approved by the Medical Ethics Committee of Shanghai Changzheng Hospital. All animal experiments used in this experiment comply with the 3Rs principle and ARRIVE guidelines. And all methods in this experiment were conducted in accordance with relevant guidelines and regulations.

### Statistical analysis

Data were analyzed using SPSS version 26.0 (IBM, USA). Data following a normal distribution were expressed as mean ± standard deviation and analyzed using Student’s *t*-test. Chi-square tests were used for categorical data comparisons. A *p*-value < 0.05 was considered statistically significant.

## Results

### KEGG pathway analysis

**Fig. 1:**
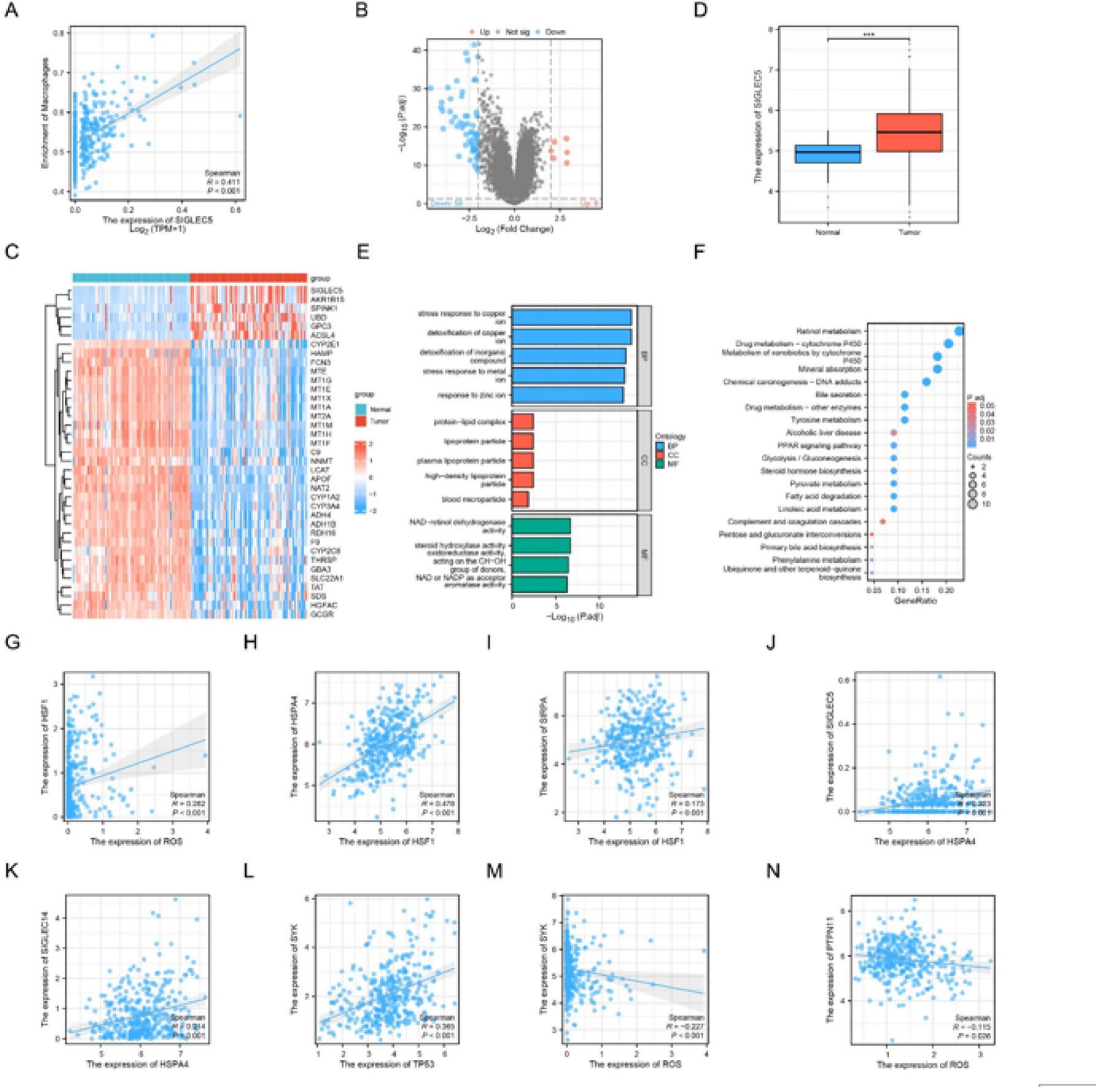
Bioinformatics analysis. (**A**) Scatter plot showing Siglec-5 immune infiltration; (**B**) Volcano plot for differential analysis; (**C**) Heatmap for differential analysis; (**D**) Expression of Siglec-5 in normal liver tissue versus hepatocellular carcinoma tissue; (**E**) Bar plot of GO enrichment analysis; (**F**) Bubble plot of KEGG enrichment analysis; (**G**) Scatter plot of ROS and HSF1 correlation; (**H**) Scatter plot of HSF1 and HSP70 (HSPA4) correlation; (**I**) Scatter plot of HSF1 and SIRPA correlation; (**J**) Scatter plot of HSP70 (HSPA4) and Siglec-5 correlation; (**K**) Scatter plot of HSP70 (HSPA4) and SIGLEC14 correlation; (**L**) Scatter plot of P53 (TP53) and SYK correlation; (**M**) Scatter plot of ROS and SYK correlation; (**N**) Scatter plot of ROS and SHP2 (PTPN11) correlation.

Differential analysis using the limma package identified 64 differentially expressed genes (DEGs), including 6 significantly upregulated genes and 58 significantly downregulated genes. A scatter plot illustrating SIGLEC5 immune infiltration. Among these, Siglec-5 was identified as a significantly upregulated gene. Data from TCGA-LIHC revealed high expression of Siglec-5 in hepatocellular carcinoma tissues. GO analysis showed that DEGs were enriched in high-density lipoprotein particle, plasma lipoprotein particle, and lipoprotein particle within Cellular Component; in steroid hydroxylase activity, aromatase activity, and NAD-retinol dehydrogenase activity within Molecular Function; and in detoxification of copper ion, stress response to copper ion, and detoxification of inorganic compound within Biological Process. KEGG pathway analysis revealed significant enrichment of DEGs in Retinol metabolism, Drug metabolism - cytochrome P450, and Mineral absorption. Correlation analysis indicated that ROS promotes the expression of HSF1, which in turn enhances the expression of HSP70 (HSPA4). HSF1 also promotes the expression of SIRPA, while HSP70 (HSPA4) promotes the expression of Siglec-5 and SIGLEC14. P53 (TP53) promotes the expression of SYK, ROS promoted the expression of SYK and ROS inhibited the expression of SHP2 (PTPN11) (Fig. 1).

### Oxidative stress due to hypoxia promoted the expression of Siglec-5 and P-SYK and inhibited the expression of P-SHP2

**Fig. 2:**
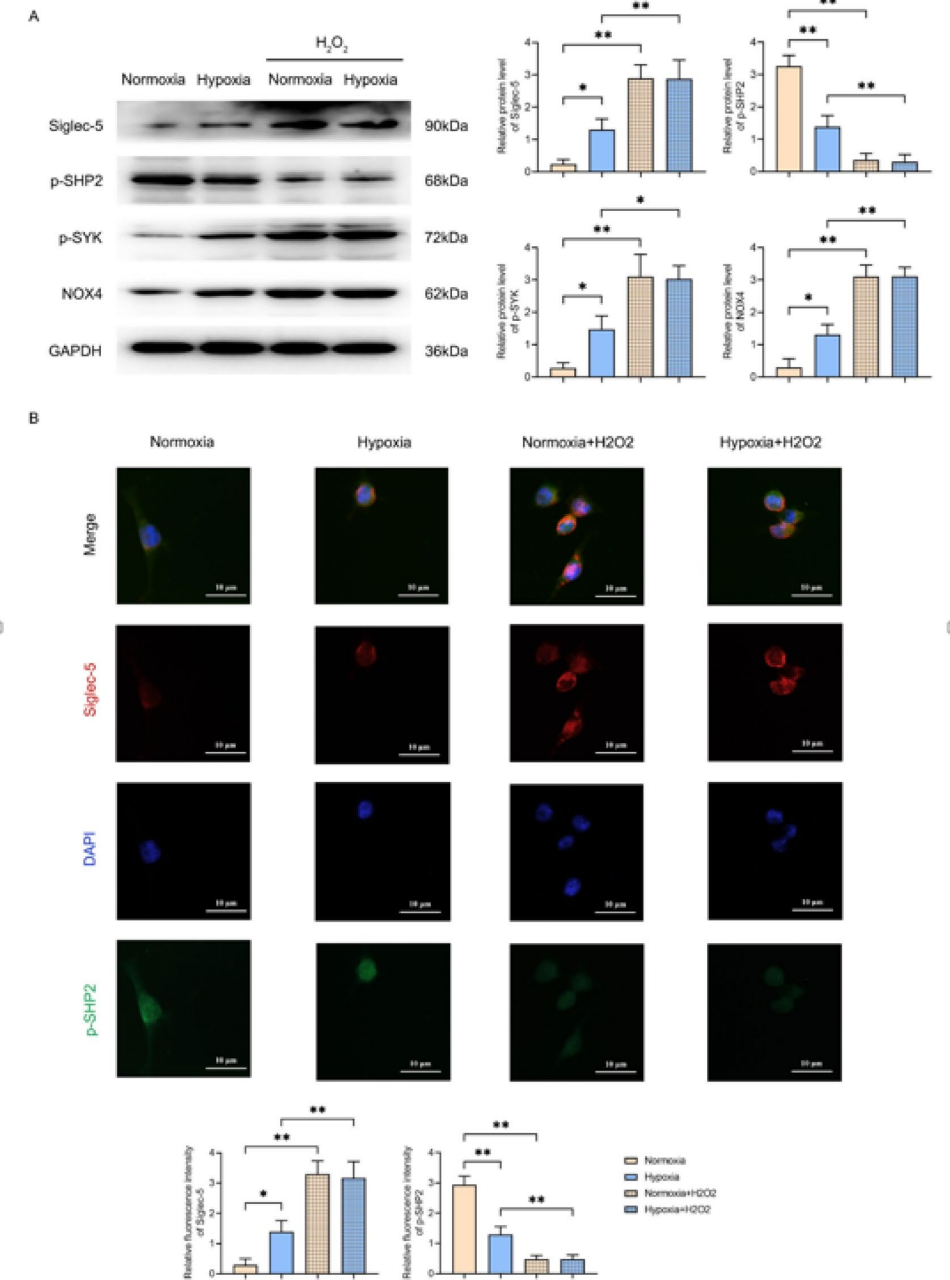
Hypoxia and oxidative stress enhance Siglec-5 expression and modulate SHP2/SYK activation in TAMs. (**A**) Western blot analysis showing protein bands and statistical plots of relative protein expression levels of Siglec-5, P-SHP2, P-SYK, and NOX4 in THP-1 macrophages in the following groups: normoxia (Group A), hypoxia (Group B), normoxia + H_2_O_2_ (Group C), and hypoxia + H_2_O_2_ (Group D); GAPDH as control protein; (**B**) Immunofluorescence staining of Siglec-5 (red) and P-SHP2 (green) in THP-1 macrophages and relative fluorescence intensity statistics. Nuclei were counterstained with DAPI (blue). Scale bar: 10 μm. Data are expressed as mean ± SD (*n* = 3). *P < 0.05; **P < 0.01.

Western blot results showed that the relative protein levels of Siglec-5, P-SYK and NOX4 were significantly higher in the hypoxia group (group B) compared with the normoxia group (group A), while the relative protein level of P-SHP2 was significantly lower. Compared with the normoxia group (group A), the relative protein levels of Siglec-5, P-SYK and NOX4 were significantly higher in the normoxia + H_2_O_2_ group (group C), while the relative protein level of P-SHP2 was significantly lower. Compared with the hypoxia group (group B), the relative protein levels of Siglec-5, P-SYK and NOX4 were significantly increased in the hypoxia + H_2_O_2_ group (group D), while the relative protein level of P-SHP2 was significantly decreased (Fig. 2A). Bioassay analysis revealed that Siglec-5 was significantly overexpressed in hepatocellular carcinoma tissues, and the differential gene was enriched in oxidative stress-related pathways. To verify this result, we examined Siglec-5 protein expression under hypoxia and H_2_O_2_ treatment by Western blot and found that it was significantly up-regulated, which was consistent with the Bio Letter prediction. In addition, the raw letter suggested that ROS was positively correlated with SYK, and the experimental hypoxic conditions significantly increased the P-SYK level, further supporting the role of the ROS-SYK axis in hepatocellular carcinoma.

Immunofluorescence results confirmed that the relative fluorescence intensity of Siglec-5 was significantly higher in the hypoxic group (group B) compared with the normoxia group (group A), whereas the relative fluorescence intensity of P-SHP2 was significantly lower. Compared with the normoxia group (group A), the relative fluorescence intensity of Siglec-5 in the normoxia + H_2_O_2_ group (group C) was significantly higher, while the relative fluorescence intensity of P-SHP2 was significantly lower. Compared with the hypoxia group (group B), the relative fluorescence intensity of Siglec-5 in the hypoxia + H_2_O_2_ group (group D) was significantly increased, whereas the relative fluorescence intensity of P-SHP2 was significantly decreased (Fig. 2B). The present results indicated that oxidative stress caused by hypoxia promoted the expression of Siglec-5 and P-SYK and inhibited the expression of P-SHP2.

### Siglec-5 promotes SHP2 expression and inhibits SYK phosphorylation

To investigate the effects of Siglec-5 on the SHP2 and SYK signaling pathways, we used Western blot and ELISA assays to detect relevant markers in THP-1 cells. Western blot experiments showed that the relative protein levels of Siglec-5, P-SYK, NOX4 and arginase-1 were significantly up-regulated in the hypoxia group (②) compared with those in the normoxia group (①), while the relative protein levels of P-SHP2, IL-1β and TNFα were significantly down-regulated. Compared with the hypoxia group (②), the relative protein levels of Siglec-5, P-SHP2, and arginase-1 were significantly elevated in the hypoxia + Siglec-5 OE group (③), whereas the relative protein levels of P-SYK, NOX4, IL-1β, and TNFα were significantly decreased.

**Fig. 3:**
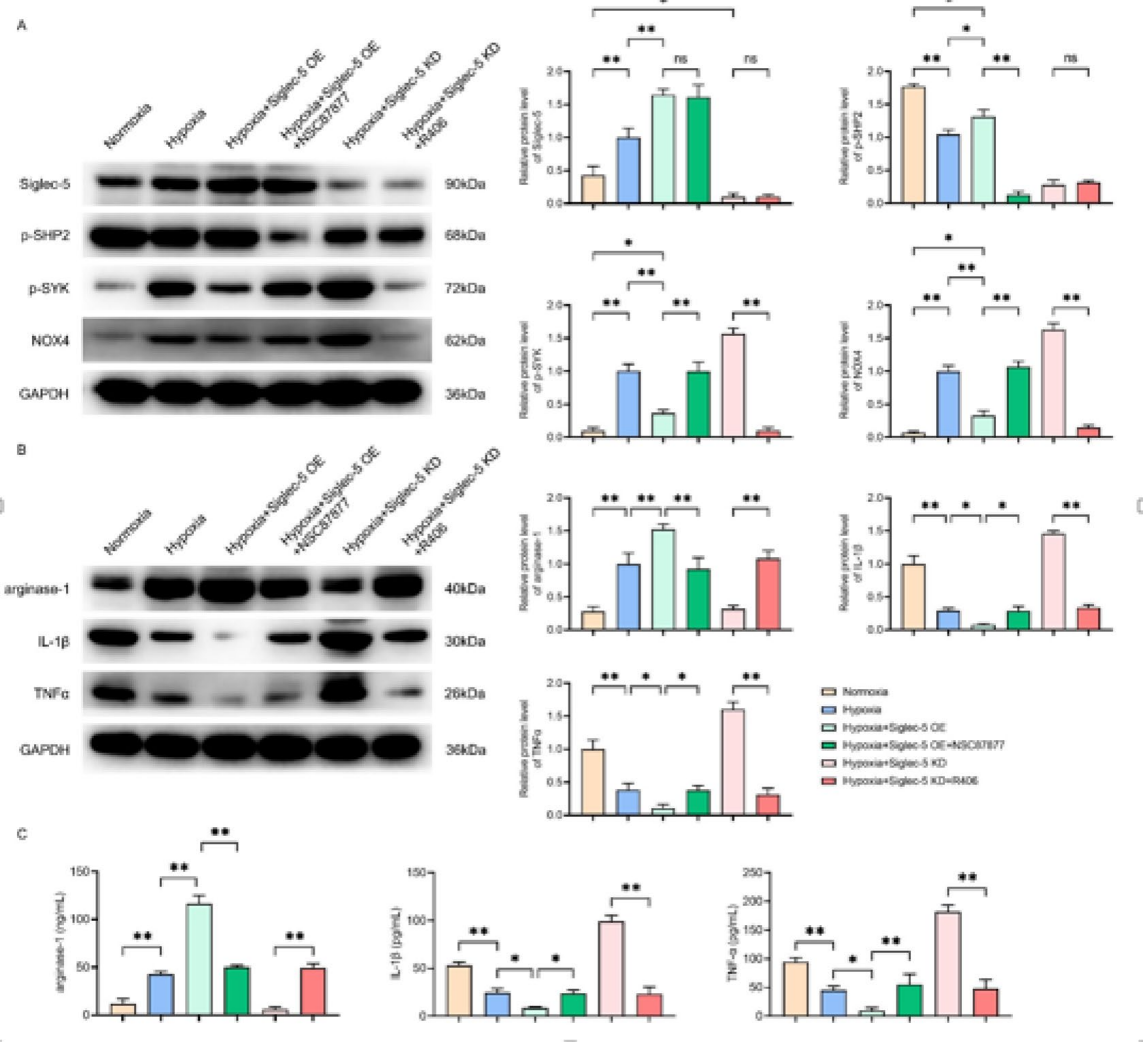
Siglec-5 regulates SHP2/SYK signaling and promotes M2 polarization and tumor progression in tumor-associated macrophages. (**A**) Western blot analysis showing protein bands and statistical plots of relative protein expression levels of Siglec-5, P-SHP2, P-SYK, and NOX4 in THP-1 macrophages in six groups: normoxia (①), hypoxia (②), hypoxia + Siglec-5 OE (③), hypoxia + Siglec-5 OE + NSC87877 (④), hypoxia + Siglec-5 KD (⑤), and hypoxia + Siglec-5 KD + R406 (⑥), (**B**) Western blot analysis showing protein bands and statistical plots of relative protein expression levels of arginase-1, IL-1β, and TNFα protein expression in THP-1 macrophages in the six groups. GAPDH as control protein; (**C**) ELISA assay measuring levels of arginase-1, IL-1β, and TNFα in THP-1 macrophages in the six groups. Data are expressed as mean ± SD (*n* = 3). *P < 0.05; **P < 0.01.

In the hypoxia + Siglec-5 OE + NSC87877 group (④), compared with the hypoxia + Siglec-5 OE group (③), there was no significant difference in the relative protein levels of Siglec-5, and there were significant decreases in the relative protein levels of P-SHP2 and arginase-1, and significant increases in the relative protein levels of P-SYK, NOX4, IL-1β and TNFα. The relative protein levels of Siglec-5 and P-SHP2 in the hypoxia + Siglec-5 KD + R406 group (6) were not significantly different from those in the hypoxia + Siglec-5 KD group (5). However, the relative protein levels of arginase-1 were significantly increased, while those of P-SYK, NOX4, IL-1β and TNFα were significantly decreased (Fig. 3A, B).

ELISA results showed that compared with the normal group (①), the level of arginase-1 was significantly increased in the hypoxia group (②), while the levels of IL-1β and TNFα were significantly decreased. In the hypoxia + Siglec-5 OE group (③), compared with the hypoxia group (②), the level of arginase-1 was significantly increased, and the levels of IL-1β and TNFα were significantly decreased. In the hypoxia + Siglec-5 OE + NSC87877 group (④), the levels of arginase-1 were significantly lower, and the levels of IL-1β and TNFα were significantly higher compared with those in the hypoxia + Siglec-5 OE group (③). In the hypoxia + Siglec-5 KD + R406 group (⑥), the levels of arginase-1 were significantly increased, and the levels of IL-1β and TNFα were significantly decreased compared with those in the hypoxia + Siglec-5 KD group (⑤) (Fig. 3C). These results indicated that Siglec-5 promoted M2 polarization of tumor-associated macrophages (TAMs) and inhibited M1 polarization by enhancing SHP2 activity while suppressing SYK phosphorylation under hypoxic conditions.

### Siglec-5 overexpression enhances hepatocellular carcinoma cell proliferation and migration under hypoxia conditions

**Fig. 4:**
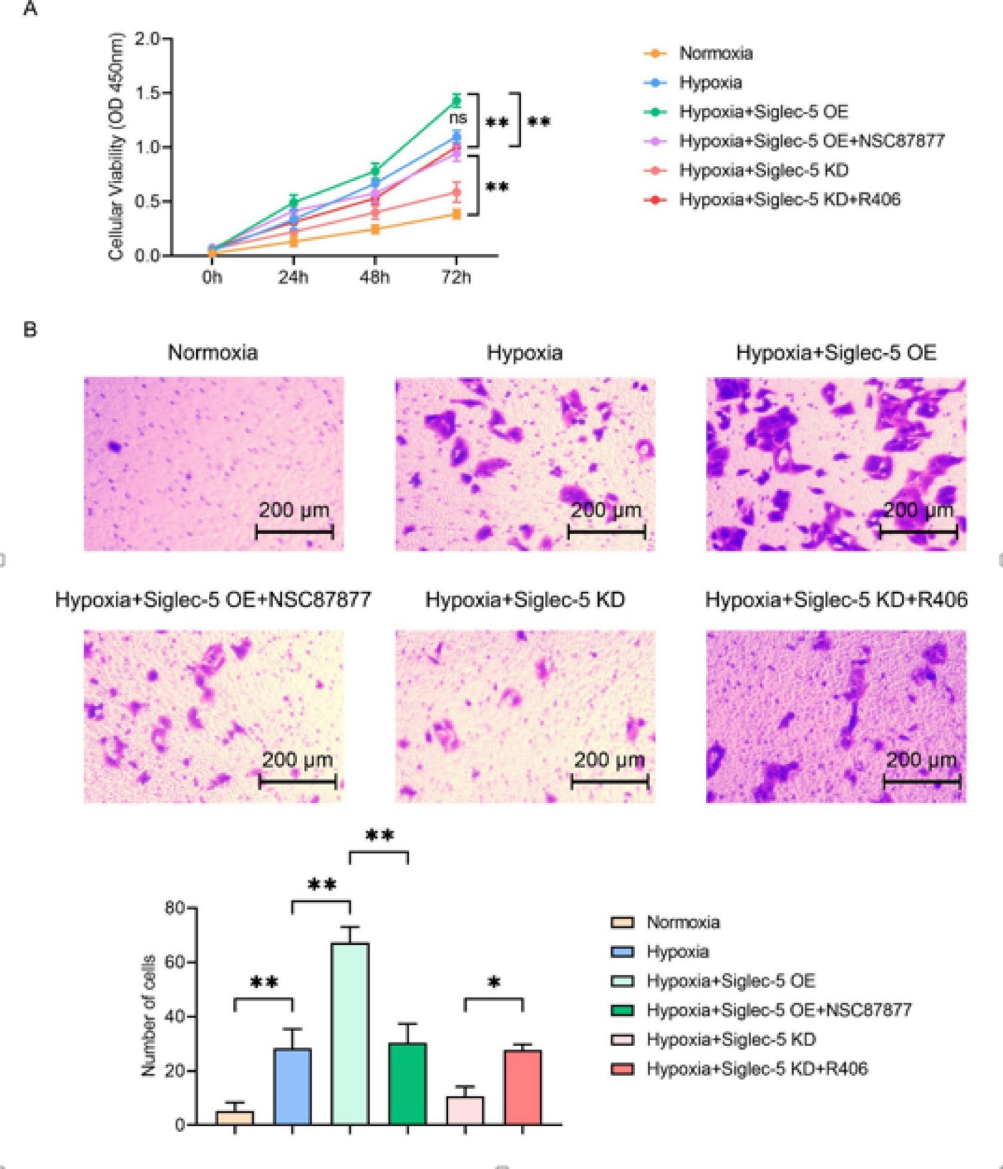
Hypoxia and Siglec-5 overexpression promote proliferation and migration of hepatocellular carcinoma cells. (**A**) CCK-8 assay evaluating hepatocellular carcinoma cell proliferation in HepG2 cells in the six groups: normoxia (①), hypoxia (②), hypoxia + Siglec-5 OE (③), hypoxia + Siglec-5 OE + NSC87877 (④), hypoxia + Siglec-5 KD (⑤), and hypoxia + Siglec-5 KD + R406 (⑥), (**B**) Transwell migration assay assessing hepatocellular carcinoma cell migration in HepG2 cells in the six groups. Scale bar: 200 μm. Data are expressed as mean ± SD (*n* = 3). *P < 0.05; **P < 0.01.

To investigate the effect of Siglec-5 on the proliferation and migration capacity of hepatocellular carcinoma cells under hypoxic conditions, we used CCK-8 and transwell assays to assess the proliferation and migration capacity of HepG2 cells. The results of CCK-8 and transwell migration assay showed that the OD value and the number of migrating cells of HepG2 cells were increased in the hypoxia group (②) compared with the normoxia group (①). The OD value and the number of migrating cells of HepG2 cells in the hypoxia + Siglec-5 OE group (③) were further increased compared with those in the hypoxia group (②). The OD value and the number of migrating cells of HepG2 cells in the hypoxia + Siglec-5 OE + NSC87877 group (④) were significantly decreased compared with the hypoxia + Siglec-5 OE group (③). Compared with the hypoxia + Siglec-5 KD group (⑤), the hypoxia + Siglec-5 KD + R406 group (⑥) showed a significant increase in the OD value and the number of migrating cells of HepG2 cells (Fig. 4). The above results indicated that Siglec-5 overexpression promotes the proliferation and migration of hepatocellular carcinoma cells under hypoxic conditions.

### Siglec-5 overexpression enhances hepatocellular carcinoma cell tumorigenicity under hypoxia conditions

**Fig. 5:**
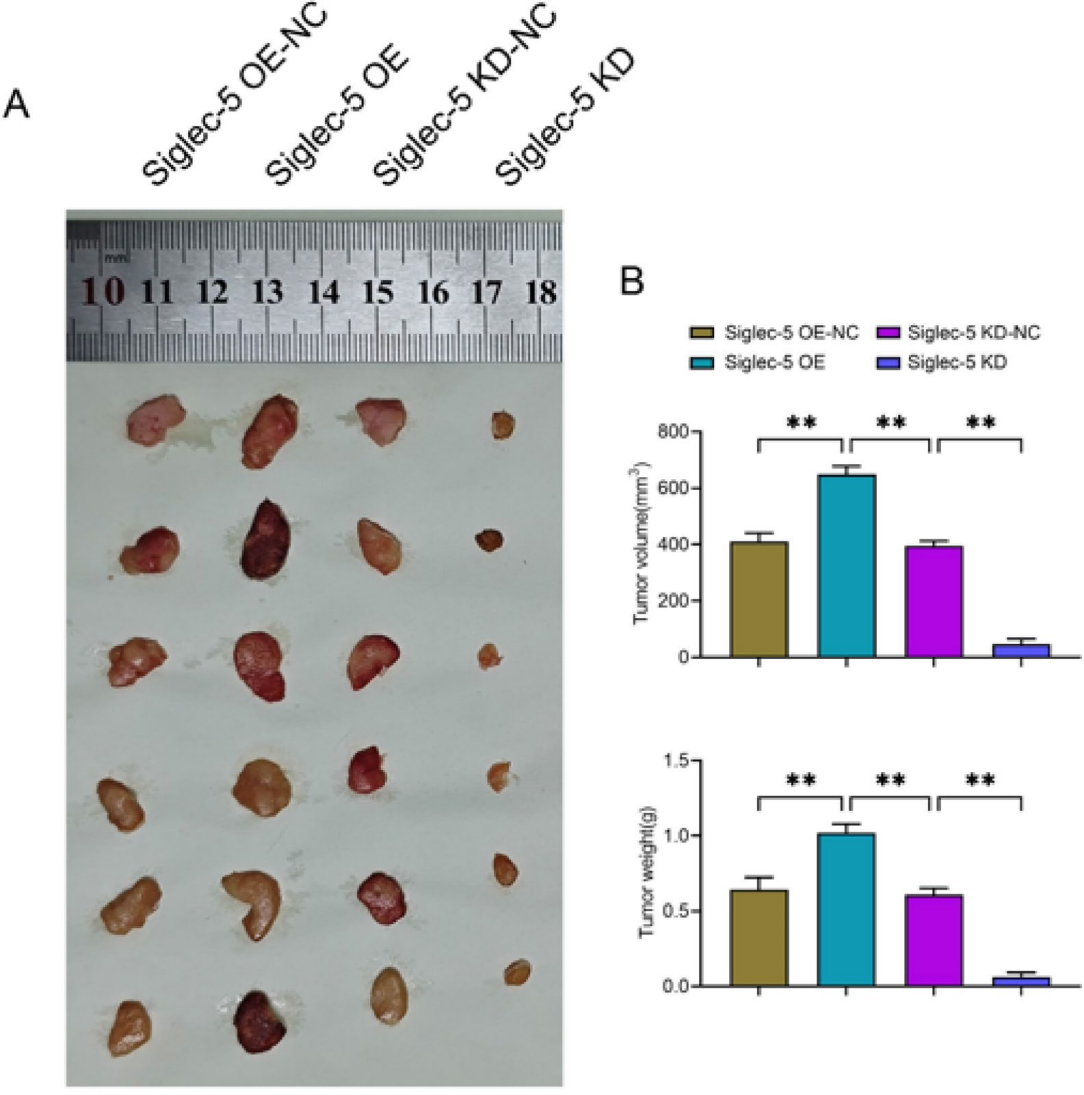
Hypoxia and Siglec-5 overexpression promote tumorigenicity of hepatocellular carcinoma cells. (**A**) Graph showing the results of subcutaneous transplantation tumor experiments in nude mice in the four groups: Sigelc-5 OE-NC gourp, Sigelc-5 OE gourp, Sigelc-5 KD-NC gourp and Sigelc-5 KD gourp, (**B**) Tumor volume and weight statistics of subcutaneous graft tumor experiments in nude mice. Data are expressed as mean ± SD (*n* = 6). *P < 0.05; **P < 0.01.

**Fig. 6:**
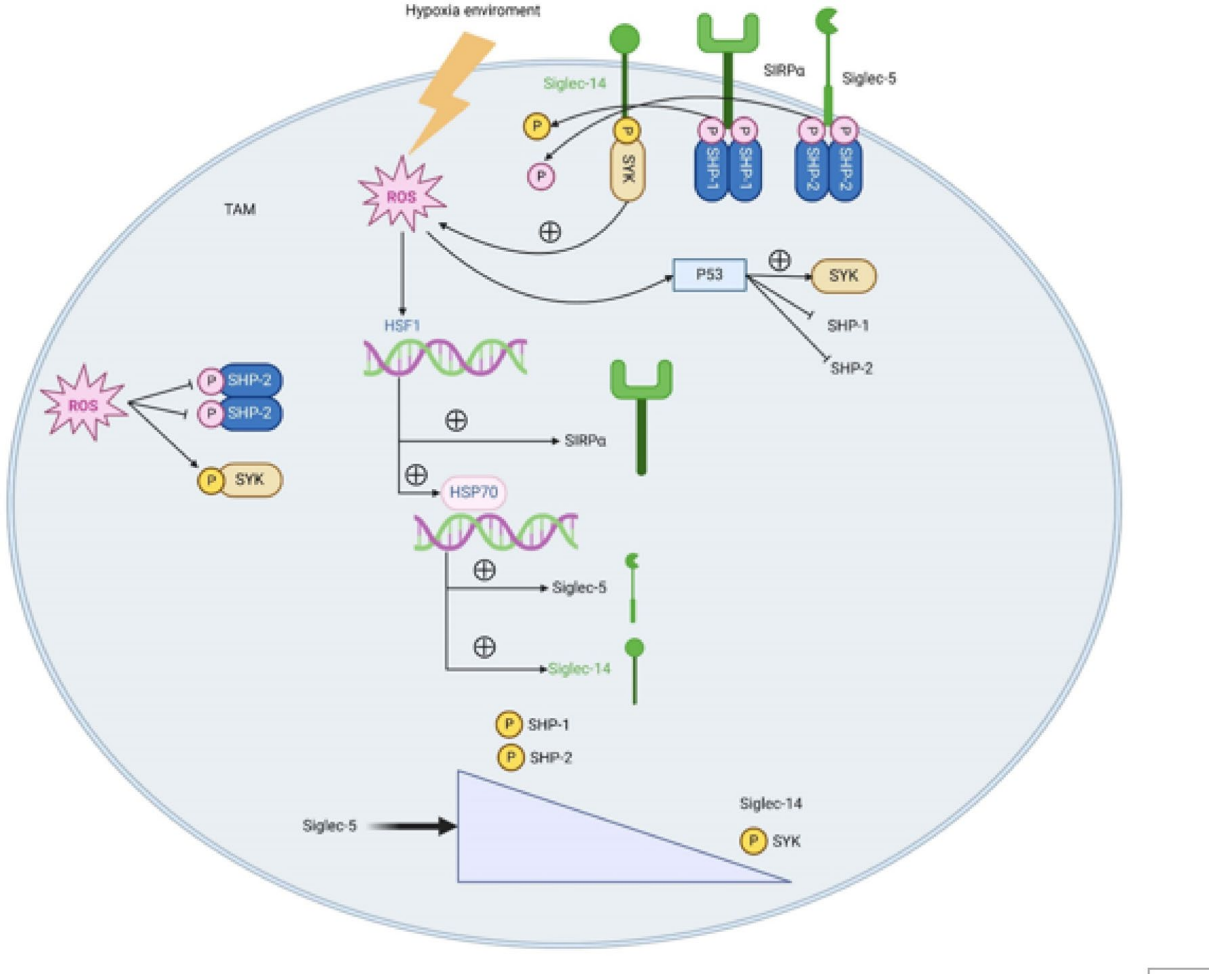
Suppressing the signaling effect of Siglec-5 in tumor-associated macrophages (TAMs) by regulating the balance of SHP2/SYK activation in hepatocellular carcinoma under hypoxia conditions. Under hypoxic conditions, the accumulation of ROS activates the HSF1/HSP70 axis, thereby upregulating the expression of Siglec-5. The activation of Siglec-5 further recruits SHP2, leading to increased phosphorylation of SHP2, which in turn inhibits the phosphorylation of SYK. Inhibiting SHP2 can relieve the inhibitory effect of Siglec-5 on SYK. This imbalance in SHP2/SYK leads to upregulation of Arg-1 expression and downregulation of IL-1β and TNFα, thereby driving the polarization of M2-type macrophages. This polarized state further promotes the proliferation, migration, and tumorigenic capacity of HepG2 cells. (The schematic diagram depicts the content occurring in tumor-associated macrophages TAMs).

Finally, we used a subcutaneous transplantation tumor assay in nude mice to verify the effect of Siglec-5 on hepatocellular carcinoma progression. The results showed that tumor volume and weight were significantly higher in the Siglec-5 OE group relative to the Siglec-5 OE-NC group. Relative to the Siglec-5 KD-NC group, tumor volume and weight were significantly lower in the Siglec-5 KD group (Fig. 5). In summary, hypoxic conditions disrupt the SHP2/SYK activation balance, which directly suppresses Siglec-5 signaling in TAMs (Fig. 6).

## Discussion

Hepatocellular carcinoma is one of the most prevalent and deadly malignant tumors worldwide. In recent years, researchers have increasingly recognized the crucial role of various molecular mechanisms within the tumor microenvironment in the progression of hepatocellular carcinoma^15^. Specifically, members of the sialic acid-binding immunoglobulin-like lectin (Siglec) family, signaling molecules such as SHP1, SHP2, and SYK, and oxidative stress-related factors like ROS play key roles in the initiation and development of hepatocellular carcinoma. Under hypoxia conditions, the complex interactions among these molecules further influence the progression of hepatocellular carcinoma. In this study, we have screened Siglec-5 as a potential key molecule in hepatocellular carcinoma by bioinformatics and predicted its association with oxidative stress and immune infiltration. Experimental validation showed that hypoxia upregulated Siglec-5 through ROS axis, activated SHP2 and inhibited SYK phosphorylation, which was highly consistent with the oxidative stress pathway and correlation analysis (ROS-SYK, HSF1-Siglec-5) enriched by biosignatures. This two-way validation strategy of raw letters guiding experiments and experiments feeding raw letters provides multidimensional evidence for mechanism resolution.

The Siglec family is a group of sialic acid-binding proteins predominantly expressed on immune cells, playing a role in regulating immune responses. Siglec-5 and Siglec-14 are paired receptors with essentially identical extracellular binding domains but differing intracellular signaling pathways^16^. In tumor-associated macrophages (TAMs), Siglec-5 associates with the tyrosine phosphatases SHP-1 and SHP-2 to transmit inhibitory signals, while Siglec-14 interacts with the adapter protein tyrosine kinase SYK to convey activating signals^6,17^. In hepatocellular carcinoma, the expression levels and functions of Siglec-5 and Siglec-14 are closely linked to tumor progression and immune evasion. Siglec-5 is primarily expressed on monocytes and macrophages, where it regulates immune functions through its interaction with sialic acid. In hepatocellular carcinoma, the expression of Siglec-5 may contribute to immune suppression within the tumor microenvironment by affecting macrophage polarization and function, thereby inhibiting anti-tumor immune responses. Siglec-5 signaling primarily relies on the recruitment and activation of SHP-1 and SHP-2, which dephosphorylate SYK (phosphorylated by Siglec-14 binding), thereby reducing immune cell anti-tumor activity. Siglec-14, an important member of the Siglec family, is also expressed on monocytes and macrophages. It activates downstream SYK signaling pathways, promoting ROS production^17^. ROS can cause direct oxidative damage to hepatocellular carcinoma cells and modulate signaling pathways, affecting tumor progression. By activating SYK, Siglec-14 inhibits HCC progression, possibly through ROS-mediated mechanisms.

SHP-1 and SHP-2 are crucial tyrosine phosphatases involved in regulating cellular signaling. In hepatocellular carcinoma, the phosphorylation status of SHP-1 and SHP-2 is important for regulating cell proliferation, migration, and immune responses. SHP-1 and SHP-2 regulate SYK dephosphorylation through interactions with Siglec-5 and SIRPα, influencing cell signaling pathways and hepatocellular carcinoma progression. SIRPα is a receptor predominantly expressed on macrophages and dendritic cells that inhibits phagocytosis by binding CD47^18^. SIRPα activation recruits SHP-1 and SHP-2, leading to their phosphorylation, which in turn dephosphorylates SYK and suppresses its activation, affecting immune cell anti-tumor functions^19^.

HSF1 (heat shock factor 1) is a key transcription factor involved in cellular responses to oxidative stress and heat shock. Under hypoxia conditions, ROS enhance HSF1 activity, which then regulates the expression of HSP70 (heat shock protein 70) and SIRPα^20^. HSP70, an important molecular chaperone, is involved in protein folding and repair, and also plays a role in immune regulation. Upregulation of HSP70 can influence immune cell functions and tumor progression by promoting the expression of Siglec-5 and Siglec-14^21^. Additionally, under hypoxia conditions, ROS can enhance P53 expression. As a tumor suppressor, P53 inhibits tumor progression through various mechanisms^22^. In hepatocellular carcinoma, ROS promotes P53 activity, thereby suppressing SHP-1 and SHP-2 expression while promoting SYK expression. Thus, ROS can inhibit SHP-1 and SHP-2 activation and enhance SYK phosphorylation. ROS regulates cellular signaling pathways through this mechanism, affecting hepatocellular carcinoma progression.

In summary, hypoxic conditions disrupt the SHP2/SYK activation balance, which directly suppresses Siglec-5 signaling in TAMs. The interactions among Siglec-5, Siglec-14, SIRPα, SHP1, SHP2, SYK, and ROS create a complex regulatory network in hepatocellular carcinoma. Within this network, interactions among oxidative stress, transcription factors, and signaling pathways collectively influence hepatocellular carcinoma progression. Specifically, increased ROS production under hypoxia conditions activates HSF1 and HSP70, promoting the expression of Siglec-5 and Siglec-14, which in turn affects immune cell functions and tumor progression. There is a balance between Siglec-5 and Siglec-14; when Siglec-5 activity is enhanced, Siglec-14 activity is reduced, thus promoting hepatocellular carcinoma progression. Additionally, SIRPα and Siglec-5 recruit SHP-1 and SHP-2 to inhibit SYK phosphorylation, potentially facilitating tumor immune evasion. ROS further affects hepatocellular carcinoma progression by modulating interactions between P53, SHP-1/SHP-2, and SYK. This mechanism not only deepens the understanding of the immune microenvironment of hepatocellular carcinoma, but also provides a theoretical basis for the development of combination therapies targeting the Siglec-5/SHP2/SYK axis. Future studies need to focus on translational applications, such as personalized therapeutic strategies based on patients’ Siglec-5 expression levels, or designing bispecific antibodies to block both Siglec-5 and PD-1/PD-L1 pathways, with the aim of breaking through the bottleneck of drug resistance in hepatocellular carcinoma treatment. However, the generalizability of the results needs to be verified in multiple hepatocellular carcinoma cell lines, primary TAMs and in vivo models in the future. In addition, further validation of the findings in patients’ hepatocellular carcinoma tissues is still needed.

## Supplementary Information

Below is the link to the electronic supplementary material.

Supplementary Material 1

## Data Availability

The datasets generated during and/or analysed during the current study are available from the corresponding author on reasonable request.
