# Supplementary material for: Hypoxia suppressed the Siglec-5 signaling in TAMs via modulating the balance of SHP2/SYK activation in hepatocellular carcinoma
Source: Sci Rep. 2025 Aug 26;15:31409. doi: 10.1038/s41598-025-14040-w (PMC12381118; doi:10.1038/s41598-025-14040-w)

Fig2A

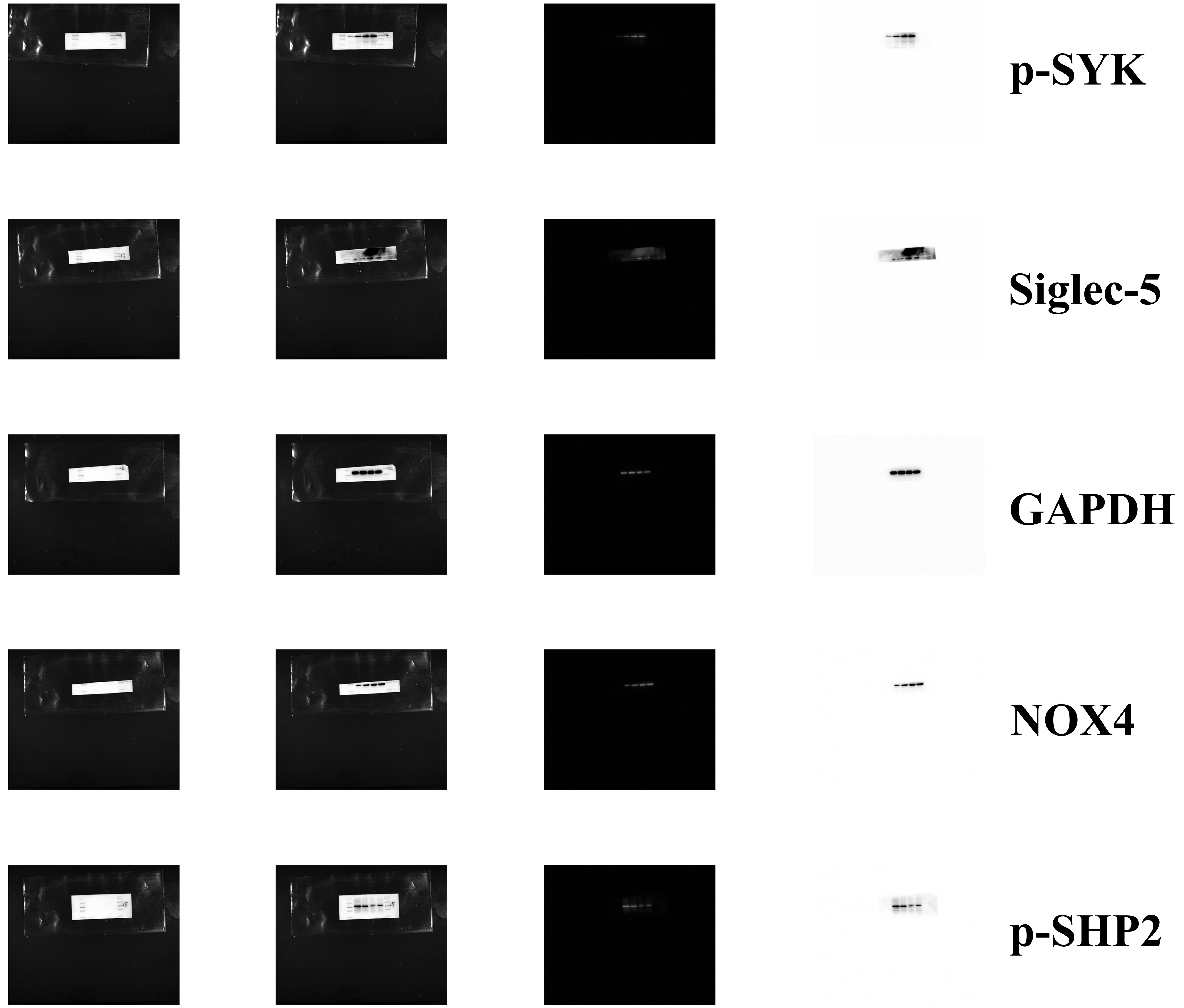

Fig3A

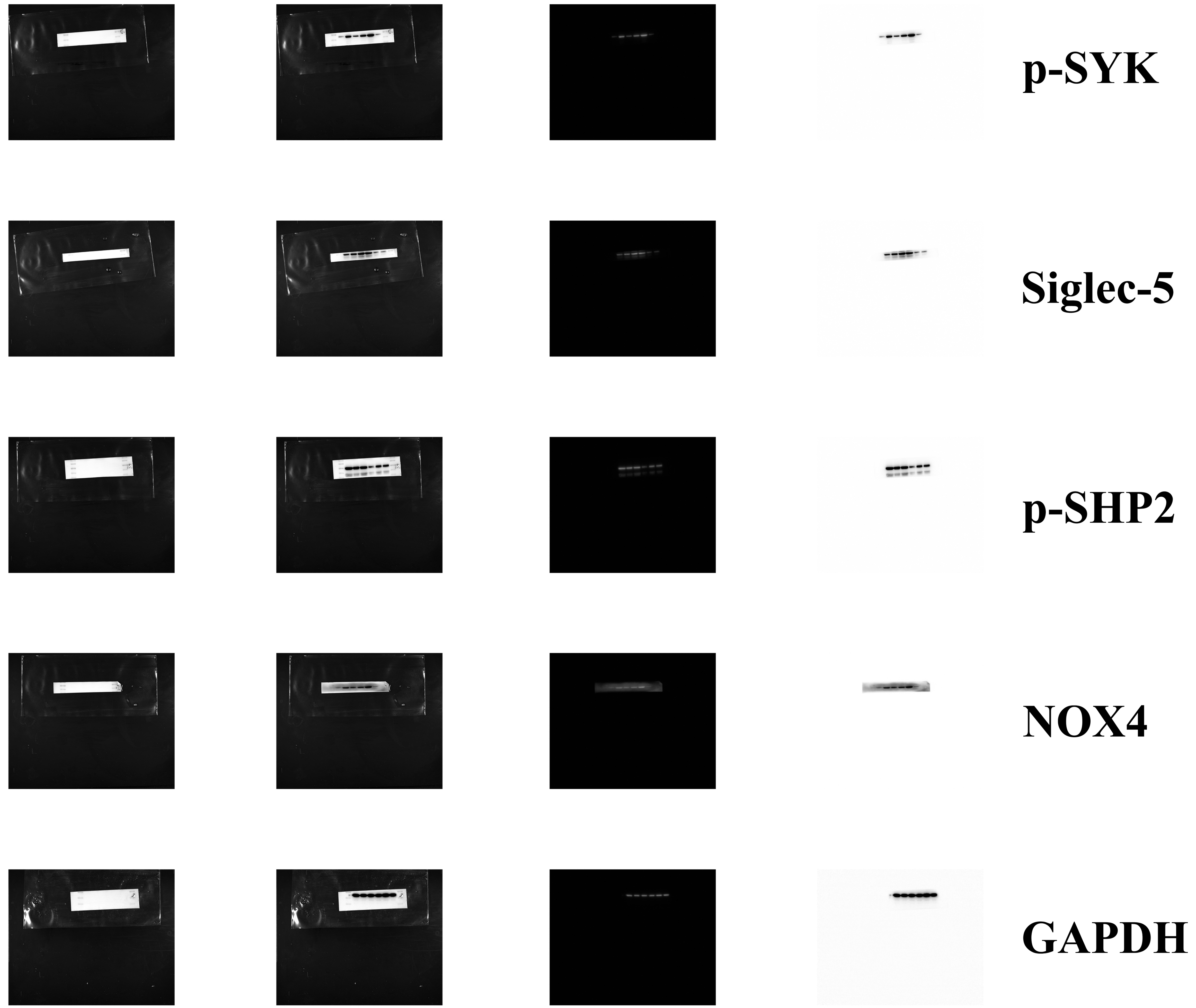

Fig3B

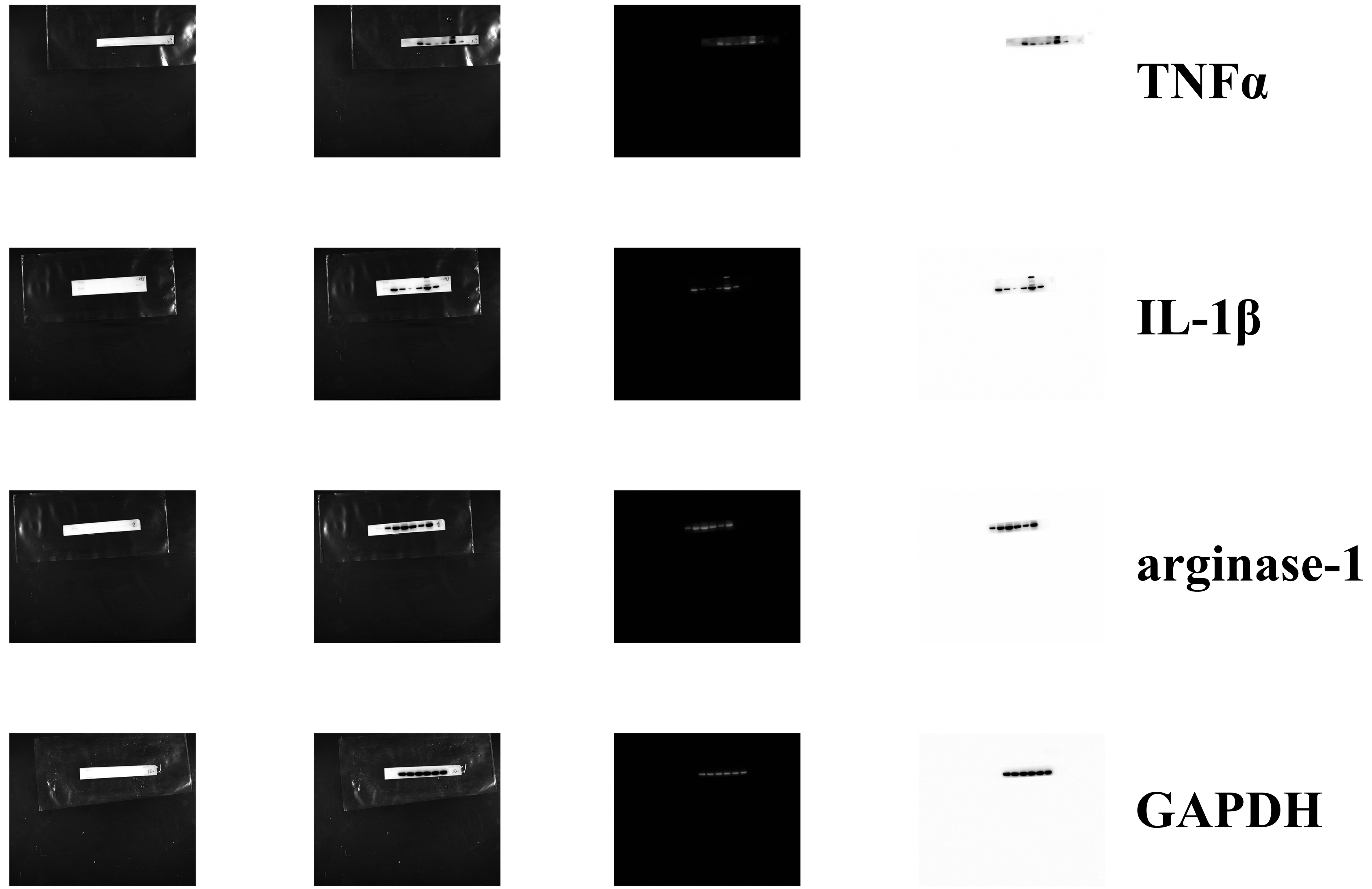

Supplement: Supplementary file 1 — Supplementary Material 1 [file 41598_2025_14040_MOESM1_ESM.pdf]
